# Optic neuritis: a comprehensive review of current therapies and emerging treatment strategies

**DOI:** 10.3389/fneur.2025.1605075

**Published:** 2025-06-18

**Authors:** Pareena Chaitanuwong, Heather E. Moss

**Affiliations:** ^1^Ophthalmology Department, Rajavithi Hospital, Ministry of Public Health, Bangkok, Thailand; ^2^Department of Ophthalmology, College of Medicine, Rangsit University, Bangkok, Thailand; ^3^Department of Ophthalmology, Stanford University, Palo Alto, CA, United States; ^4^Department of Neurology & Neurological Sciences, Stanford University, Palo Alto, CA, United States

**Keywords:** optic neuritis, multiple sclerosis, corticosteroids, treatment in optic neuritis, emerging therapies

## Abstract

**Background:**

Optic neuritis (ON) is an inflammatory condition of the optic nerve that can lead to significant visual impairment. It is often associated with multiple sclerosis (MS) but can also occur in other demyelinating diseases, such as neuromyelitis optica spectrum disorder (NMOSD) and myelin oligodendrocyte glycoprotein antibody disease (MOGAD). Understanding the current therapeutic approaches and emerging treatment strategies is critical for optimizing patient outcomes.

**Objective:**

This review provides a focused overview of current therapies for demyelinating optic neuritis associated with MS, NMOSD, and MOGAD. Less common autoimmune optic neuropathies, non-autoimmune causes (e.g., infections) and pediatric optic neuritis are not covered.

**Methods:**

A review of the literature was conducted, including clinical trials, observational studies, and expert recommendations on the treatment and management of demyelinating ON. The efficacy, safety, and limitations of various therapeutic modalities were assessed.

**Results:**

High-dose intravenous corticosteroids remain the mainstay of acute demyelinating ON treatment, accelerating visual recovery but not altering long-term visual outcomes. Immunomodulatory therapies, such as disease-modifying treatments for MS, play a crucial role in preventing recurrent episodes in demyelinating diseases. Emerging therapies, including re-myelination agents, neuroprotective strategies, and novel immunotherapies, show promise in improving visual prognosis and reducing long-term disability.

**Conclusion:**

While corticosteroids remain the primary treatment for acute demyelinating ON, ongoing research into neuroprotective and re-myelinating therapies offers hope for better visual recovery and long-term management. Future studies should focus on optimizing treatment strategies and exploring novel therapeutics to enhance patient outcomes.

## 1 Introduction

Optic neuritis (ON) is a demyelinating disorder of the optic nerve that can lead to sudden vision loss in one or both eyes. Optic neuritis can be caused by both infectious and non-infectious factors. The most common non-infectious cause of optic neuritis worldwide is multiple sclerosis (MS) (1), but it can also occur in association with other demyelinating diseases, such as neuromyelitis optica spectrum disorder (NMOSD) and myelin oligodendrocyte glycoprotein antibody-associated disease (MOGAD), or without an identifiable cause. The relative incidence of different causes varies according to geographic location.

The standard treatment for optic neuritis is intravenous methylprednisolone (IVMP) (2), which has been shown to hasten the recovery of vision but not impact long-term visual outcome of MS and idiopathic forms. In recent years, there has been a growing interest in the use of other treatments for optic neuritis, such as oral steroids, plasma exchange, and other immunomodulatory drugs.

This review focuses on current treatment strategies for demyelinating ON in the context of MS, NMOSD, and MOGAD. Rare autoimmune [e.g., Chronic Relapsing Inflammatory Optic Neuritis (CRION), Autoimmune glial fibrillary acid protein (GFAP), autoimmune collapsin response-mediator protein-5 (CRMP5)], non-autoimmune causes (e.g., infectious ON) and pediatric optic neuritis are beyond the scope of this article. Furthermore, we will discuss the potential future directions of research in the field of optic neuritis treatment.

## 2 Overview of autoimmune optic neuritis

Autoimmune ON is primarily categorized into two types: typical ON (idiopathic or MS-associated) and atypical ON. The common atypical ON cases, characterized by biomarkers such as aquaporin-4 (AQP4) and myelin oligodendrocyte glycoprotein (MOG) antibodies, represents distinct CNS demyelinating diseases. Less common autoimmune optic neuropathies such as CRION, GFAP-associated astrocytopathy, and paraneoplastic CRMP5 optic neuropathy, are differentiated by clinical presentation and testing. Differentiating between typical and atypical ON is essential for understanding prognosis and treatment variations. While the Optic Neuritis Treatment Trial (ONTT) (3) remains foundational in guiding the acute treatment of typical optic neuritis, several limitations must be acknowledged in light of evolving understanding. Notably, the study was conducted prior to the identification of biomarkers for NMOSD and MOGAD. Furthermore, patients with bilateral, recurrent or severe ON phenotypes were excluded. As a result, its findings are not generalizable to NMOSD or MOGAD-associated optic neuritis. In addition, the ONTT was predominantly composed of young white females from North America, limiting its applicability to more diverse populations. The emergence of serological testing and advanced MRI biomarkers has significantly refined ON diagnostic and prognostic stratification, necessitating an updated framework for interpreting ONTT results in today's clinical practice (3).

### 2.1 Typical optic neuritis (MS-associated, idiopathic)

Typical ON usually presents with unilateral vision loss in young adults and tends to recover spontaneously. While most patients regain good visual acuity, MRI and CSF analysis are important for assessing MS risk. Recent diagnostic criteria updates now allow earlier MS diagnosis in ON patients, which may influence early therapeutic decisions (4–6).

### 2.2 Atypical optic neuritis (NMO, MOG)

Atypical ON is often bilateral, more severe, and recurrent, with poorer visual prognosis. NMOSD-associated ON is linked with AQP4-IgG and often involves the optic chiasm (7). MOGAD-associated ON is typically seen in younger patients and presents with marked disc edema and good recovery, though relapses are not common (8, 9). Accurate diagnosis of both conditions via antibody testing and/or clinical criteria is essential, as treatment differs from typical ON (10, 11).

As our understanding of optic neuritis expands, especially with the identification of atypical forms such as NMOSD and MOGAD, accurate classification has become increasingly important for guiding management. These subtypes differ markedly in clinical presentation, diagnostic pathways, and therapeutic approaches. Due to overlapping features and evolving diagnostic criteria, clinicians may face challenges in early differentiation. To support clinical decision-making, Table 1 provides a visual summary that contrasts typical and atypical ON, highlighting key diagnostic clues and outlining distinct treatment strategies for demyelinating ON. Management of optic neuritis can be challenging due to decisions for treatment being required prior to availability of supporting evidence (MRI, NMO serology, MOG serology) to triage as to the underlying cause. In these situations clinical features can assist in preliminary triage as MS/idiopathic, NMO-SD (severe vision loss, bilateral involvement) and MOG (severe disc edema). It is particularly important to be alert for features of NMO-SD so that therapy can be escalated accordingly.

**Table 1:** Simplified clinical and therapeutic approach to typical vs. atypical optic neuritis.

**Category**	**Typical (MS-associated/idiopathic)**	**Atypical (NMO-SD)**	**Atypical (MOGAD)**
Clinical features	- Young adult - Usually Unilateral - Mild-moderate vision loss - Usually no optic nerve head swelling - Short optic nerve enhancement on MRI - Good spontaneous recovery	- Adult - Unilateral or bilateral - Severe vision loss - Mild or no optic nerve head swelling - Long posterior optic nerve enhancement on MRI - Poor spontaneous recovery	- Pediatric and adult - Unilateral or bilateral - Mild–severe vision loss - Optic nerve head usually swelling - Long anterior optic nerve with perineural enhancement on MRI - Variable spontaneous recovery
Acute treatment	High-dose steroids with short taper	High-dose steroids with long taper + PLEX	High-dose steroids with long taper Add PLEX if severe or refractory
Disease diagnosis	Clinical criteria + MRI +/– CSF oligoclonal band	Serum AQP4 IgG with clinical criteria	Serum MOG IgG with clinical criteria
Long term maintenance	MS DMTs	Early immunotherapy avoid MS DMTs	Immunotherapy in patients with severe or relapsing disease MS DMTs is not effective

MS, multiple sclerosis; NMOSD, neuromyelitis optica spectrum disorder; MOGAD, myelin oligodendrocyte glycoprotein associated disease; MRI, magnetic resonance imaging; CSF, cerebrospinal fluid; IVMP, intravenous methylprednisolone; PLEX, plasma exchange; AQP4, aquaporin 4; IgG, immunoglobulin G; DMTs, disease modifying therapies.

## 3 Treatment approaches for optic neuritis and review of clinical studies

### 3.1 Acute treatment

High-dose corticosteroids, either oral or intravenous (IV), are the primary treatments for acute autoimmune ON. The largest trial, ONTT, found that high dose intravenous corticosteroids (1 g/day for 3 consecutive days) expedite initial visual recovery within the first 2 weeks, by about 1–2 lines of Snellen visual acuity. Interestingly, low-dose oral prednisone (1 mg/kg) was associated with increased risk of ON relapse within the initial 2 years, discouraging its use. Recent studies have shown that high-dose oral corticosteroids may be non-inferior to high-dose intravenous steroid treatments for treating MS relapses (12). Additionally, high-dose oral methylprednisolone may be a cost-effective alternative with comparable patient satisfaction to traditional intravenous administration (13).

Notably, ONTT results may not be broadly generalizable, especially for atypical ON variants like NMOSD-ON. Acute flares of NMOSD-ON, including optic neuritis, should be treated with IVMP (1 g/day for 3–5 consecutive days with or without a PO prednisone taper). Early treatment has been shown to correlate with preservation of peripapillary retinal nerve fiber layer. Retrospective studies suggest that IV corticosteroids alone might be suboptimal for visual recovery in NMOSD-ON, and that concurrent or sequential plasma exchange (PLEX) may improve outcomes. PLEX can be initiated in IVMP-refractory disease, with one series reporting average final visual acuity of 20/50 in NMOSD-ON patients receiving sequential IVMP and PLEX compared to 20/400 in those receiving IVMP alone. In two non-randomized studies of acute NMOSD (including some ON cases), 40%−50% of attacks treated with PLEX within 2 days of symptom onset experienced complete recovery, and 0%−5% recovering fully with PLEX initiation after 20 days (14, 15). Although high-quality randomized controlled trial data specifically addressing the impact of PLEX in NMOSD are currently lacking, retrospective studies suggest potential benefits from early PLEX therapy when combined with high-dose corticosteroids.

In cases of MOGAD, much like with other ON, the standard approach to acute treatment typically involves IVMP, which tends to yield rapid responses in most patients (16, 17). One retrospective study encompassing both AQP4-IgG+NMOSD and MOGAD cases found a potential benefit from initiating treatment at an earlier stage (18). For individuals experiencing severe attacks with significant disability at the peak of the attack, it is advisable to consider the early implementation of a combined therapy involving intravenous corticosteroids and plasma exchange (PLEX) (19).

Several studies have evaluated non-steroid treatments for acute optic neuritis, but none have shown a clear benefit (Table 2). Some of the failure to demonstrate benefit might relate to trial design. Future trials should prioritize earlier administration post-injury, focus on well-characterized patient subgroups with features likely to benefit from the intervention (e.g., active demyelination but limited neurodegeneration in the case of re-myelinating agents), and adopt more sensitive and specific outcome measures, such as functional imaging and electrophysiological assessments targeting affected white matter tracts. Due to varying recovery patterns in different ON subtypes (NMOSD, MOG, etc.), expert-opinion-based treatment approaches are being proposed.

**Table 2:** Research on non-steroid treatments for acute optic neuritis.

**Citation**	**Intervention**	**Sample size**	**Study design**	**Result**
Roed et al. (20) Denmark	IVIG	30 treatment, 34 placebo	Single-Center, randomized controlled trial	There was no effect of IVIG on long-term visual function, nor an effect in reducing latency on VEP.
Tsakiri et al. (21) Denmark	Simvastatin 80 mg	32 treatment, 32 placebo	Single-Center, randomized controlled trial	Simvastatin had a beneficial effect on VEP in both latency and amplitude, a borderline effect on the Arden score and Visual analog scale, and no effect on brain MRI or on relapse rate between the groups.
Suhs et al. (22) Germany	33,000 IU recombinant human erythropoietin intravenously daily for 3 days—add-on treatment with IVMP	20 treatment, 17 placebo	Single-center, phase 2, randomized controlled trial	Testing of visual functions at 16 weeks follow up revealed trends toward an improved outcome after erythropoietin treatment.
Raftopoulos et al. (23) UK	Phenytoin 4–6 mg/kg/day 3 months—after treating with IVMP	39 treatment, 42 placebo	Multi-center, phase 2, randomized controlled trial	At 6 months follow up, 30% reduction in the extent of RNFL loss with phenytoin compared with placebo.
Cadavid et al. (24) Australia	Opicinumab 100 mg/kg intravenous once weekly for 6 dose—after treating with IVMP	33 treatment, 36 placebo	Multi-center, phase 2, randomized controlled trial	Remyelination did not differ significantly between the opicinumab and placebo groups at week 24.
McKee et al. (25) UK	Amiloride 10 mg/day 5 months—after treating with IVMP	22 treatment, 26 placebo	Multi-center, phase 2, randomized controlled trial	Amiloride has not demonstrated any neuroprotective benefit within this trial.
Falardeau et al. (26) USA	Oral lipoic acid 1,200 mg/day 6 weeks—after treating with IVMP	15 treatment, 16 placebo	Single center, randomized controlled trial	No conclusion that lipoic acid treatment was neuroprotective in acute optic neuritis
Albert et al. (27) Germany	0.5 mg oral fingolimod daily VS subcutaneous IFN-β 1b 250 μg every other day. For 6 months - after treating with IVMP	6 Fingolimod 7 IFN-β 1b	Multi-Center, randomized controlled trial	Not enough data to conclude.

IVIG, intravenous immunoglobulin; VEP, visual evoked potential; MRI, magnetic resonance imaging; IVMP, intravenous methylprednisolone; RNFL, retinal nerve fiber layer; IFN-β 1b, interferon beta 1 B.

### 3.2 Long term management

Prevention of recurrence and preservation of long-term visual function are the primary objectives of long-term treatment following acute optic neuritis. The prognosis following the initial attack, the likelihood of relapses and the appropriate treatment are contingent upon the underlying inflammatory condition (i.e., MS, NMO or MOG). When a diagnosis of MS, NMO or MOG cannot be established it is important to reassess the patient on a regular basis to determine if diagnostic criteria have been met so that appropriate treatment can be initiated.

Optic neuritis associated with NMOSD typically exhibits the poorest visual prognosis from the outset, and subsequent relapses can worsen this. In NMOSD, extended courses of oral prednisone are typically given following acute therapy [intravenous methylprednisolone (IVMP) with or without PLEX], with consideration for early initiation of immunosuppressive disease modifying therapy to reduce risk of future episodes of optic neuritis.

Similarly, MOGAD presents a less favorable visual prognosis compared to multiple sclerosis (MS) optic neuritis. After the administration of IVMP, patients are often started on an oral prednisone tapering regimen, which typically extends beyond the two-week duration recommended by the ONTT, and consideration of PLEX for severe cases with poor recovery. Disease modifying therapy initiation in MOGAD is based on relapses and persistence of serum MOG antibodies.

Multiple sclerosis (MS) treatment encompasses a comprehensive array of strategies aimed at managing the disease's complex facets. Many FDA-approved medications have demonstrated efficacy in managing MS over the long term. Disease-modifying therapy (DMT) forms the cornerstone of management of MS by reducing relapse frequency and neurological disability. These therapies have evolved over time, with newer options replacing earlier, less targeted immunosuppressive treatments. Treatment strategies vary, with escalation from low efficacy to high efficacy medications. However, a newer approach involves initiating higher-efficacy treatments at diagnosis to achieve better relapse control.

#### 3.2.1 Multiple sclerosis DMT

MS disease modifying therapies act by modulating the immune system through diverse mechanisms, including sequestration of lymphocytes, alteration of cytokine secretion patterns, and immune cell depletion and vary by efficacy, adverse effects and route of administration (Table 3). Newly developed DMTs, including monoclonal antibodies exhibit heightened efficacy compared to traditional oral and injectable options. While infusion reactions and autoimmune side effects remain potential concerns, their overall benefits are significant.

**Table 3:** The table categorizes the FDA-approved disease-modifying therapies (DMTs) for multiple sclerosis (MS) by their method of administration (29).

**Category**	**Medications**
Self-injectable	Glatiramer acetate (Copaxone^®^, Glatopa^®^), Interferon beta-1a (Avonex^®^, Betaseron^®^, Extavia^®^, Rebif^®^), Peginterferon beta-1a (Plegridy^®^), Ofatumumab (Kesimpta^®^)
Oral	Cladribine (Mavenclad^®^), Dimethyl fumarate (Tecfidera^®^), Diroximel fumarate (Vumerity^®^), Fingolimod (Gilenya^®^, Tascenso ODT^®^), Monomethyl fumarate (Bafiertam™), Ozanimod (Zeposia^®^), Ponesimod (Ponvory™), Siponimod (Mayzent^®^), Teriflunomide (Aubagio^®^)
Infused	Alemtuzumab (Lemtrada^®^), Mitoxantrone (Novantrone^®^), Natalizumab (Tysabri^®^) Natalizumab-sztn (Tyruko^®^), Ocrelizumab (Ocrevus^®^), Ublituximab-xiiy (Briumvi™)

Selection of specific MS DMT are tailored to individual patient's needs. Although DMT treatment is typically long-term, people with stable disease while on certain DMTs may choose to de-escalate therapy. The reader is referred to the excellent review by McGinley et al. for more information (28).

#### 3.2.2 NMOSD

Certain drugs that are approved for multiple sclerosis (MS) treatment should not be used in patients with neuromyelitis optica spectrum disorder (NMOSD) (30, 31), especially in those who are AQP4-IgG-positive. Some of these drugs, such as glatiramer acetate, are simply ineffective in preventing NMOSD attacks (32). Others, such as interferon beta (31), natalizumab (33), fingolimod, alemtuzumab (34), and dimethyl fumarate (35), have been reported to trigger severe NMOSD attacks.

Mitoxantrone may have some effect on reducing the frequency of NMOSD attacks, but it should no longer be used due to its unfavorable safety profile and the limited duration of treatment (36). Cyclophosphamide is another drug that has been tried in NMOSD, but the results have been conflicting. It is not recommended for use in NMOSD due to the limited total dose allowance and potentially severe side effects (37).

Many studies have been conducted in the last few years to develop medications to treat NMOSD. These new drugs have shown significant promise in clinical trials, reducing the frequency of relapses and improving neurological function in NMOSD patients. As a result, four new drugs have been approved in the U.S. for the treatment of NMOSD: eculizumab, inebilizumab, satralizumab, and ravulizumab- cwvz.

- Eculizumab is a monoclonal antibody that blocks the complement protein C5. It is the only drug approved for both AQP4-IgG seropositive and AQP4-IgG seronegative NMOSD (38).
- Inebilizumab is a monoclonal antibody that targets CD19-positive B cells. It is approved for the treatment of AQP4-IgG seropositive NMOSD (39).
- Satralizumab is a monoclonal antibody that targets the interleukin-6 receptor. It is approved for the treatment of AQP4-IgG seropositive NMOSD as an adjunct therapy to other immunosuppressants or as monotherapy (40, 41).
- Ravulizumab-cwvz is a long-acting C5 complement inhibitor. It is approved for the treatment of adult patients with AQP4-IgG seropositive NMOSD (42).

In addition to these new drugs, other immunosuppressants, such as azathioprine, mycophenolate mofetil, and rituximab, are also commonly used to treat NMOSD based on historical experience. However, these drugs are not FDA-approved for NMOSD. The choice of treatment for NMOSD should be individualized based on the patient's clinical presentation, serologic status, and response to previous treatments. Additional information can be found in the excellent review by Gospe et al. (43).

#### 3.2.3 MOGAD

Long-term management of myelin oligodendrocyte glycoprotein antibody-associated disease (MOGAD) focuses on preventing relapses and limiting cumulative neurological damage. The risk of relapse is influenced by several factors, including persistent seropositivity for MOG-IgG, especially when present at high titers, and a history of multiple clinical events. Conversely, pediatric patients (44), male patients presenting with spinal cord involvement and those who receive corticosteroid therapy for at least 1 month during the first attack are more likely to experience a monophasic disease course and a longer time to first relapse (45). Additionally, seroconversion to negative MOG-IgG has been associated with a reduced risk of recurrence (45). About half of people with MOGAD only have one relapse, and those who do have relapses often make a good recovery after the first one (46). Some experts advise that patients with MOGAD who make a full recovery after the first attack do not need to take long-term immunosuppressive drugs until they are proven to have relapsing disease by having another relapse (47).

Currently, there are no FDA approved treatments for MOGAD. Disease-modifying MS medications, such as interferon-β and glatiramer acetate have been shown to be ineffective in MOGAD, while data on natalizumab are ambiguous (25). In contrast, immunosuppressants such as mycophenolate mofetil (MMF), azathioprine, intravenous immunoglobulin (IVIG), and rituximab have all been associated with reductions in the annualized relapse rate (ARR) (48, 49). A recent multi-center retrospective study found that IVIG was associated with the lowest relapse rate among a variety of immunosuppressants (50). Furthermore, a recent meta-analysis of 41 primarily retrospective observational studies demonstrated that azathioprine, MMF, rituximab, IVIG, and tocilizumab are effective in reducing relapse risk in both pediatric and adult patients with MOGAD (51). Despite these promising findings, it is important to note that, to date, no randomized controlled trials have been conducted for MOGAD, underscoring the urgent need for high-quality prospective studies to guide evidence-based treatment strategies.

## 4 Future directions in optic neuritis treatment

Current treatments for optic neuritis primarily aim to reduce acute inflammation and prevent long-term relapses, but they are not always effective. There is an urgent need for new and more effective treatments for optic neuritis. Some of the most promising future directions in optic neuritis treatment include:

- Neuroprotective treatments that aim to prevent damage to the optic nerve during inflammatory attacks. Promising agents include memantine, erythropoietin, interferon-beta, phenytoin, and clemastine. However, recent literature reviews have not yet demonstrated significant clinical differences, and further studies are needed in the future (52).
- Remyelination therapies that promote the repair of damaged myelin in the optic nerve. Notable remyelinating agents under clinical investigation for treating optic neuritis in multiple sclerosis (MS) include Ibudilast and Mesenchymal Stem Cells (MSC) (53).
  Among remyelination-promoting therapies, opicinumab, a monoclonal antibody targeting LINGO1, a CNS protein that inhibits remyelination, was developed based on preclinical studies showing promotion of oligodendrocyte survival, axonal regeneration, and remyelination. Phase I trials confirmed its safety, leading to three Phase II studies: RENEW (optic neuritis) (24), SYNERGY (MS) (54), and AFFINITY (MS). While RENEW showed delayed improvement in visual evoked potentials, none of the trials met their primary endpoints, and development was discontinued following the failure of AFFINITY (55). Despite promising results in animal models, the human clinical translation of opicinumab has been limited by challenges such as blood-brain barrier penetration, identification of reliable biomarkers, and determining the optimal treatment window (56). Early intervention, ideally within 25 days of symptom onset, and careful patient selection appear critical, with better responses seen in older patients with optic neuritis and younger MS patients with preserved CNS structure (24, 54, 55).
- Gene therapy is a rapidly advancing field with the potential to develop regenerative treatments for optic neuritis and other optic neuropathy. One promising approach involves delivering neuroprotective gene-encoding proteins directly into the optic nerve. This strategy is currently under preclinical investigation and may also hold therapeutic potential for other neurological disorders in the future (51). Another emerging direction is gene therapy aimed at enabling or enhancing retinal ganglion cell (RGC) regeneration and functional integration (57). While this remains in the experimental stage, such therapies could ultimately transform care for patients with irreversible vision loss. Large-scale collaborative efforts, such as the RGC Repopulation, Stem Cell Transplantation, and Optic Nerve Regeneration (RReSTORe) Consortium, are essential to overcoming current challenges and translating these advances into clinical practice (58). For more information, the author suggests an excellent review by Esposito et al. on this topic (59).

Further research is needed to elucidate the underlying pathophysiological mechanisms of MS-, NMOSD- and MOGAD-associated optic neuritis. A better understanding of these mechanisms, may facilitate the development of newer, more precise and targeted therapies aimed at preserving vision and improving quality of life for the affected individuals.

## 5 Conclusion

In conclusion, optic neuritis is a complex condition with various underlying causes. It presents with sudden and often painful vision loss and can be associated with demyelinating diseases like multiple sclerosis (MS), neuromyelitis optica spectrum disorder (NMOSD), and myelin oligodendrocyte glycoprotein antibody disease (MOGAD). While intravenous methylprednisolone (IVMP) remains the standard treatment for acute optic neuritis of these varieties, its impact on long-term visual outcomes is limited. Therefore, exploring alternative treatments has become an area of growing interest.

Typical optic neuritis, often associated with MS, tends to have a favorable prognosis, with most patients experiencing significant visual recovery. However, monitoring and early diagnosis are crucial for identifying those at risk of developing MS. Treatment strategies for MS involve disease-modifying therapies (DMTs), which aim to reduce relapse frequency and disability.

Atypical optic neuritis, especially in the context of NMOSD and MOGAD, presents unique challenges. Differentiating between typical and atypical forms is essential, as their prognoses and treatment approaches differ significantly. NMOSD, characterized by the presence of AQP4-IgG antibodies, benefits from PLEX in the acute setting and immunosuppressive therapies like eculizumab, inebilizumab, and satralizumab in the long term. MOGAD ON can have excellent recovery and minimal risk of relapse, but some cases have poor recovery and relapsing disease. In these more severe cases PLEX and chronic treatments like mycophenolate mofetil (MMF), azathioprine, intravenous immunoglobulin (IVIG), and rituximab show promise in reducing relapse rates.

Ongoing research in neuroprotective therapies, remyelination strategies, gene therapy and disease-specific pathophysiology holds promise for the development of targeted and effective treatments for optic neuritis.

## References

[1] Toosy AT Mason DF Miller DH. Optic neuritis. Lancet Neurol. (2014) 13:83–99. 10.1016/S1474-4422(13)70259-X 24331795

[2] Beck RW Gal RL. Treatment of acute optic neuritis: a summary of findings from the optic neuritis treatment trial. Arch Ophthalmol. (2008) 126:994–5. 10.1001/archopht.126.7.994 18625951 PMC9353544

[3] Beck RW. The optic neuritis treatment trial. Arch Ophthalmol. (1988) 106:1051–3 10.1001/archopht.1988.01060140207023 3041942

[4] Thompson AJ Banwell BL Barkhof F Carroll WM Coetzee T Comi G. Diagnosis of multiple sclerosis: 2017 revisions of the McDonald criteria. Lancet Neurol. (2018) 17:162–73 10.1016/S1474-4422(17)30470-2 29275977

[5] Montalban. Revisions of the McDonald Criteria. Scientific Session 1: New diagnostic Criteria. Denmark: ECTRIMS Congress Copenhagen (2024).

[6] Huang J Khademi M Fugger L Lindhe Ö Novakova L Axelsson M. Inflammation-related plasma and CSF biomarkers for multiple sclerosis. Proc Natl Acad Sci U S A. (2020) 117:12952–60. 10.1073/pnas.1912839117 32457139 PMC7293699

[7] Winter A Chwalisz B. MRI characteristics of NMO, MOG and MS related optic neuritis. Semin Ophthalmol. (2020) 35:333–42. 10.1080/08820538.2020.1866027 33395326

[8] Jurynczyk M Messina S Woodhall MR Raza N Everett R Roca-Fernandez A. Clinical presentation and prognosis in MOG-antibody disease: a UK study. Brain. (2017) 140:3128–38. 10.1093/brain/awx276 29136091

[9] Cobo-Calvo A Ruiz A Maillart E Audoin B Zephir H Bourre B. Clinical spectrum and prognostic value of CNS MOG autoimmunity in adults: the MOGADOR study. Neurology. (2018) 90:e1858–e69. 10.1212/WNL.0000000000005560 29695592

[10] Wingerchuk DM Banwell B Bennett JL Cabre P Carroll W Chitnis T. International consensus diagnostic criteria for neuromyelitis optica spectrum disorders. Neurology. (2015) 85:177–89. 10.1212/WNL.0000000000001729 26092914 PMC4515040

[11] Banwell B Bennett JL Marignier R Kim HJ Brilot F Flanagan EP. Diagnosis of myelin oligodendrocyte glycoprotein antibody-associated disease: international MOGAD panel proposed criteria. Lancet Neurol. (2023) 22:268–82. 10.1016/S1474-4422(22)00431-8 36706773

[12] Burton JM O'Connor PW Hohol M Beyene J. Oral versus intravenous steroids for treatment of relapses in multiple sclerosis. Cochrane Database Syst Rev. (2012) 12:Cd006921. 10.1002/14651858.CD006921.pub3 23235634 PMC12145957

[13] Horta-Hernández AM Esaclera-Izquierdo B Yusta-Izquierdo A Martín-Alcalde E Blanco-Crespo M Álvarez-Nonay A. High-dose oral methylprednisolone for the treatment of multiple sclerosis relapses: cost-minimisation analysis and patient's satisfaction. Eur J Hosp Pharm. (2019) 26:280–4. 10.1136/ejhpharm-2018-001499 31656616 PMC6788278

[14] Kleiter I Gahlen A Borisow N Fischer K Wernecke KD Hellwig K. Apheresis therapies for NMOSD attacks: a retrospective study of 207 therapeutic interventions. Neurol Neuroimmunol Neuroinflamm. (2018) 5:e504. 10.1212/NXI.0000000000000504 30345331 PMC6192689

[15] Bonnan M Valentino R Debeugny S Merle H Fergé JL Mehdaoui H. Short delay to initiate plasma exchange is the strongest predictor of outcome in severe attacks of NMO spectrum disorders. J Neurol Neurosurg Psychiatry. (2018) 89:346–51. 10.1136/jnnp-2017-316286 29030418

[16] Marignier R Hacohen Y Cobo-Calvo A Pröbstel AK Aktas O Alexopoulos H. Myelin-oligodendrocyte glycoprotein antibody-associated disease. Lancet Neurol. (2021) 20:762–72. 10.1016/S1474-4422(21)00218-0 34418402

[17] Ramanathan S Mohammad S Tantsis E Nguyen TK Merheb V Fung VSC. Clinical course, therapeutic responses and outcomes in relapsing MOG antibody-associated demyelination. J Neurol Neurosurg Psychiatry. (2018) 89:127–37. 10.1136/jnnp-2017-316880 29142145 PMC5800335

[18] Stiebel-Kalish H Hellmann MA Mimouni M Paul F Bialer O Bach M. Does time equal vision in the acute treatment of a cohort of AQP4 and MOG optic neuritis? Neurology Neuroimmunol Neuroinflamm. (2019) 6:e572. 10.1212/NXI.0000000000000572 31355308 PMC6624092

[19] Weinshenker BG O'Brien PC Petterson TM Noseworthy JH Lucchinetti CF Dodick DW. A randomized trial of plasma exchange in acute central nervous system inflammatory demyelinating disease. Ann Neurol. (1999) 46:878–86. 10.1002/1531-8249(199912)46:6<878::aid-ana10>3.0.co;2-q 10589540

[20] Roed H Langkilde A Sellebjerg F Lauritzen M Bang P Mørup A. A double-blind, randomized trial of IV immunoglobulin treatment in acute optic neuritis. Neurology. (2005) 64:804–10. 10.1212/01.WNL.0000152873.82631.B3 15753413

[21] Tsakiri A Kallenbach K Fuglø D Wanscher B Larsson H Frederiksen J. Simvastatin improves final visual outcome in acute optic neuritis: a randomized study. Mult Scler J. (2012) 18:72–81. 10.1177/1352458511415452 21921071

[22] Sühs KW Hein K Sättler MB Görlitz A Ciupka C Scholz K. A randomized, double-blind, phase 2 study of erythropoietin in optic neuritis. Ann Neurol. (2012) 72:199–210. 10.1002/ana.23573 22926853

[23] Raftopoulos R Hickman SJ Toosy A Sharrack B Mallik S Paling D. Phenytoin for neuroprotection in patients with acute optic neuritis: a randomised, placebo-controlled, phase 2 trial. Lancet Neurol. (2016) 15:259–69. 10.1016/S1474-4422(16)00004-1 26822749

[24] Cadavid D Balcer L Galetta S Aktas O Ziemssen T Vanopdenbosch L. Safety and efficacy of opicinumab in acute optic neuritis (RENEW): a randomised, placebo-controlled, phase 2 trial. Lancet Neurol. (2017) 16:189–99. 10.1016/S1474-4422(16)30377-5 28229892

[25] McKee JB Cottriall CL Elston J Epps S Evangelou N Gerry S. Amiloride does not protect retinal nerve fibre layer thickness in optic neuritis in a phase 2 randomised controlled trial. Mult Scler J. (2019) 25:246–55. 10.1177/1352458517742979 29172994

[26] Falardeau J Fryman A Wanchu R Marracci GH Mass M Wooliscroft L. Oral lipoic acid as a treatment for acute optic neuritis: a blinded, placebo controlled randomized trial. Mult Scler J Exp Transl Clin. (2019) 5:2055217319850193. 10.1177/2055217319850193 31205740 PMC6537072

[27] Albert C Mikolajczak J Liekfeld A Piper SK Scheel M Zimmermann HG. Fingolimod after a first unilateral episode of acute optic neuritis (MOVING) – preliminary results from a randomized, rater-blind, active-controlled, phase 2 trial. BMC Neurol. (2020) 20:1–12. 10.1186/s12883-020-01645-z 32126977 PMC7052969

[28] McGinley MP Goldschmidt CH Rae-Grant AD. Diagnosis and treatment of multiple sclerosis: a review. JAMA. (2021) 325:765–79. 10.1001/jama.2020.26858 33620411

[29] Wingerchuk DM Carter JL. Multiple sclerosis: current and emerging disease-modifying therapies and treatment strategies. Mayo Clinic Proc. (2014) 89:225–40. 10.1016/j.mayocp.2013.11.002 24485135

[30] Barnett MH Sutton I. Neuromyelitis optica: not a multiple sclerosis variant. Curr Opin Neurol. (2012) 25:215–20. 10.1097/WCO.0b013e3283533a3f 22487568

[31] Shimizu J Hatanaka Y Hasegawa M Iwata A Sugimoto I Date H. IFNβ-1b may severely exacerbate Japanese optic-spinal MS in neuromyelitis optica spectrum. Neurology. (2010) 75:1423–7. 10.1212/WNL.0b013e3181f8832e 20826711

[32] Ayzenberg I Schöllhammer J Hoepner R Hellwig K Ringelstein M Aktas O. Efficacy of glatiramer acetate in neuromyelitis optica spectrum disorder: a multicenter retrospective study. J Neurol. (2016) 263. 10.1007/s00415-015-7991-1 26810718

[33] Kornberg MD Newsome SD. Unmasking and provoking severe disease activity in a patient with NMO spectrum disorder. Neurol Neuroimmunol Neuroinflamm. (2015) 2:e66. 10.1212/NXI.0000000000000066 25738165 PMC4335815

[34] Kowarik MC Hoshi M Hemmer B Berthele A. Failure of alemtuzumab as a rescue in a NMOSD patient treated with rituximab. Neurol Neuroimmunol Neuroinflamm. (2016) 3:e208. 10.1212/NXI.0000000000000208 26913290 PMC4751152

[35] Javadian N Magrouni H Ghaffarpour M Ranji-Burachaloo S. Severe relapses of neuromyelitis optica spectrum disorder during treatment with dimethyl fumarate. Clin Neuropharmacol. (2021) 44:21–2. 10.1097/WNF.0000000000000430 33449476

[36] Enriquez CAG Espiritu AI Pasco PMD. Efficacy and tolerability of mitoxantrone for neuromyelitis optica spectrum disorder: a systematic review. J Neuroimmunol. (2019) 332:126–34. 10.1016/j.jneuroim.2019.04.007 31005713

[37] Kageyama T Miyamoto K Ozaki A Komori M Okunomiya T Kambe D. Long-term effect of cyclosporine A in the prophylaxis of neuromyelitis optica spectrum disorders (P2.160). Neurology. (2016) 86(16 Suppl):P2.160. 10.1212/WNL.86.16_supplement.P2.16031425902

[38] Pittock SJ Berthele A Fujihara K Kim HJ Levy M Palace J. Eculizumab in aquaporin-4–positive neuromyelitis optica spectrum disorder. N Engl J Med. (2019) 381:614–25. 10.1056/NEJMoa1900866 31050279

[39] Cree BA Bennett JL Kim HJ Weinshenker BG Pittock SJ Wingerchuk DM. Inebilizumab for the treatment of neuromyelitis optica spectrum disorder (N-MOmentum): a double-blind, randomised placebo-controlled phase 2/3 trial. Lancet. (2019) 394:1352–63. 10.1016/S0140-6736(19)31817-3 31495497

[40] Traboulsee A Greenberg BM Bennett JL Szczechowski L Fox E Shkrobot S. Safety and efficacy of satralizumab monotherapy in neuromyelitis optica spectrum disorder: a randomised, double-blind, multicentre, placebo-controlled phase 3 trial. Lancet Neurol. (2020) 19:402–12. 10.1016/S1474-4422(20)30078-8 32333898 PMC7935419

[41] Yamamura T Kleiter I Fujihara K Palace J Greenberg B Zakrzewska-Pniewska B. Trial of satralizumab in neuromyelitis optica spectrum disorder. N Engl J Med. (2019) 381:2114–24. 10.1056/NEJMoa1901747 31774956

[42] Pittock SJ Barnett M Bennett JL Berthele A de Sèze J Levy M. Ravulizumab in aquaporin-4-positive neuromyelitis optica spectrum disorder. Ann Neurol. (2023) 93:1053–68. 10.1002/ana.26626 36866852

[43] Gospe SM Chen JJ Bhatti MT. Neuromyelitis optica spectrum disorder and myelin oligodendrocyte glycoprotein associated disorder-optic neuritis: a comprehensive review of diagnosis and treatment. Eye. (2021) 35:753–68. 10.1038/s41433-020-01334-8 33323985 PMC8026985

[44] Bruijstens AL Breu M Wendel E-M Wassmer E Lim M Neuteboom RF. E.U. paediatric MOG consortium consensus: part 4 – outcome of paediatric myelin oligodendrocyte glycoprotein antibody-associated disorders. Eur J Paediatr Neurol. (2020) 29:32–40. 10.1016/j.ejpn.2020.10.007 33183945

[45] Huda S Whittam D Jackson R Karthikeayan V Kelly P Linaker S. Predictors of relapse in MOG antibody associated disease: a cohort study. BMJ Open. (2021) 11:e055392. 10.1136/bmjopen-2021-055392 34848526 PMC8634280

[46] Han JY Kim SY Kim W Kim H Cho A Choi J. Phenotype of relapsing myelin oligodendrocyte glycoprotein antibody-associated disease in Children. J Clin Neurol. (2025) 21:65–73. 10.3988/jcn.2024.0276 39778568 PMC11711267

[47] Matteo G Thomas F Giacomo G Silvia S Eleonora R Stefano M. Prognostic relevance of quantitative and longitudinal MOG antibody testing in patients with MOGAD: a multicentre retrospective study. J Neurol Neurosurg Psychiatry. (2023) 94:201. 10.1136/jnnp-2022-330237 36460438

[48] Chen JJ Flanagan EP Bhatti MT Jitprapaikulsan J Dubey D Lopez Chiriboga AS. Steroid-sparing maintenance immunotherapy for MOG-IgG associated disorder. Neurology. (2020) 95:e111–e20. 10.1212/WNL.0000000000009758 32554760 PMC7455322

[49] Li S Ren H Xu Y Xu T Zhang Y Yin H. Long-term efficacy of mycophenolate mofetil in myelin oligodendrocyte glycoprotein antibody-associated disorders. Neurology. (2020) 7:e705. 10.1212/NXI.0000000000000705 32170045 PMC7136046

[50] Whittam DH Karthikeayan V Gibbons E Kneen R Chandratre S Ciccarelli O. Treatment of MOG antibody associated disorders: results of an international survey. J Neurol. (2020) 267:3565–77. 10.1007/s00415-020-10026-y 32623595 PMC7954658

[51] Chang X Zhang J Li S Wu P Wang R Zhang C. Meta-analysis of the effectiveness of relapse prevention therapy for myelin-oligodendrocyte glycoprotein antibody-associated disease. Mult Scler Relat Disord. (2023) 72:104571. 10.1016/j.msard.2023.104571 36905816

[52] Tsai TH Lin CW Chan LW Tew TB Chen TC. Neuroprotective effects of novel treatments on acute optic neuritis–a meta-analysis. Biomedicines. (2022) 10:192. 10.3390/biomedicines10010192 35052875 PMC8774005

[53] Aneesh A Liu A Moss HE Feinstein D Ravindran S Mathew B. Emerging concepts in the treatment of optic neuritis: mesenchymal stem cell-derived extracellular vesicles. Stem Cell Res Ther. (2021) 12:594. 10.1186/s13287-021-02645-7 34863294 PMC8642862

[54] Cadavid D Mellion M Hupperts R Edwards KR Calabresi PA Drulović J. Safety and efficacy of opicinumab in patients with relapsing multiple sclerosis (SYNERGY): a randomised, placebo-controlled, phase 2 trial. Lancet Neurol. (2019) 18:845–56. 10.1016/S1474-4422(19)30137-1 31285147

[55] Biogen Inc. Biogen Reports Q3 2020 Results. Cambridge MA: Biogen (2020)

[56] Neumann B Foerster S Zhao C Bodini B Reich DS Bergles DE. Problems and pitfalls of identifying remyelination in multiple sclerosis. Cell Stem Cell. (2020) 26:617–9. 10.1016/j.stem.2020.03.017 32386552

[57] Harvey AR. Gene therapy and the regeneration of retinal ganglion cell axons. Neural Regen Res. (2014) 9:232–3. 10.4103/1673-5374.128213 25206805 PMC4146148

[58] Soucy JR Aguzzi EA Cho J Gilhooley MJ Keuthan C Luo Z. Retinal ganglion cell repopulation for vision restoration in optic neuropathy: a roadmap from the RReSTORe consortium. Mol neurodegener. (2023) 18:64. 10.1186/s13024-023-00655-y 37735444 PMC10514988

[59] Esposito EP Han IC Johnson TV. Chapter 15 - Gene and cell-based therapies for retinal and optic nerve disease. In:Federoff HJ Barker RA, editors. Handbook of Clinical Neurology. Vol. 205. Amsterdam: Elsevier (2024). p. 243–62. 10.1016/B978-0-323-90120-8.00016-2 39341657

